# Editorial: New perspectives in fighting multidrug-resistant organisms: Natural sources of bioactive compounds

**DOI:** 10.3389/fmicb.2023.1158958

**Published:** 2023-02-28

**Authors:** Rosanna Salvia, Carlo Genovese, Floriana D'Angeli, Patrizia Falabella

**Affiliations:** ^1^Department of Sciences, University of Basilicata, Potenza, Italy; ^2^Spinoff XFlies s.r.l, University of Basilicata, Potenza, Italy; ^3^Faculty of Medicine and Surgery, “Kore” University of Enna, Enna, Italy; ^4^Nacture S.r.l, Spin-Off University of Catania, Catania, Italy; ^5^Department of Human Sciences and Quality of Life Promotion, San Raffaele Roma Open University, Rome, Italy

**Keywords:** natural sources, bioactive compounds, multidrug-resistant organisms (MDROs), antimicrobial activity, antimicrobials

Nowadays, a large range of antimicrobial drugs are available and used to treat many human infections. In particular, antibiotics are potent drugs used to treat pathogenic bacteria. However, their extensive and indiscriminate use, especially in developing countries, in both human medical and veterinary treatment, has contributed to the development and spread of antibiotic-resistant bacteria (Antimicrobial Resistance Collaborators, 2022). The alarming global increase in resistance to conventional antibiotics poses a potential and significant risk to public health as the World Health Organization has announced. The emergence of multidrug-resistant organisms (MDROs) constitutes a serious problem for public health (Catalano et al., 2022). Over the past few decades, significant efforts have been made to study novel molecules against antibiotic-resistant microorganisms. However, throughout the past three decades, only a small number of new classes of antibiotics have become commercially available, and few are currently undergoing human clinical trials (Moretta et al., 2021). In this context, natural sources play a prominent role in the drug discovery field due to the presence of compounds able to interfere with the biological functions of different microorganisms. The natural sources of antimicrobial molecules include plants, microorganisms, essential oils, and insects, and it is on these molecules of natural origin that scientific investigations are focusing (Tanhaeian et al., 2020; Genovese et al., 2021; Li et al., 2021; Manniello et al., 2021). Some of them are reported in the research article collection of Frontiers in Microbiology published under the Research Topic “New Perspectives in Fighting Multidrug-Resistant Organisms: Natural Sources of Bioactive Compounds”. Below is an overview of the subject matters and key findings of each article included in this collection.

Traditionally, *Allium* species have been used as medicinal plants due to their antimicrobial activity. Chen et al. investigated the antimicrobial effects of root, leaf, and scape extracts of Chinese chive, an Asian popular food derived from *Allium* species. Specifically, the effect of these extracts was evaluated against different bacterial and fungal species, including *Pectobacterium carotovorum, Pseudomonas syringae, Fusarium proliferatum*, and *Alternaria brassicicola*. The whole scape extract showed better in a higher activity. The 2-amino-5-methylbenzoic acid represented the main scape extract active compound identified through the HPLC and GC–MS analysis. Besides, the authors studied the mechanism of action of Chinese chive scape extract, demonstrating that it causes changes at high relative conductivity and protein leakage, disrupting the cell wall and plasma membranes.

*Cannabis sativa* extracts, traditionally used for several medicinal purposes due to the presence of cannabinoids and terpenes, seem to demonstrate antimicrobial and antifungal activities.

Skala et al. performed three extractions with three different solvents [ethanol (EtOH), butane (BUT), and dimethyl ether (DME)], investigating their influence on the chemical profile of *C. sativa* extracts. Although EtOH extract contained fewer cannabinoids and terpenes compared to BUT and DME, all the extracts showed antimicrobial activity against eighteen out of the nineteen tested microorganisms. However, due to the lower content of cannabinoids and terpenes, EtOH extract showed a lower antimicrobial effect. In this study, bacteria and dermatophytes, responsible for various skin diseases, were used. The results obtained showed that natural extracts, traditionally used for several diseases, represent a valid alternative to commonly used antibiotics for the treatment of skin infections.

Lan et al. studied the antimicrobial and antibiofilm activities of the natural molecule Disaspidin BB against eleven clinically resistant *Staphylococcus epidermidis* strains responsible for skin infections. Interestingly, Disaspidin BB inhibited bacterial growth in a dose-dependent manner. Furthermore, Disaspidin BB exerted a scavenging effect on *S. epidermidis* biofilm at the stages of adhesion, aggregation, and maturation. To investigate the mechanism of action of Disaspidin BB on *S. epidermidis* biofilm, the effect of the natural compound on the main components of the biofilm matrix (polysaccharides, proteins, and eDNA) was measured. The authors observed a significant decrease in polysaccharides, proteins, and eDNA after exposure to Disaspidin BB. In particular, a further molecular investigation showed that Disaspidin BB inhibited biofilm formation by affecting the expression levels of key genes (aap, atlE, icaA, luxS, recA) in *S. epidermidis* biofilm formation. This study contributes to adding a possible alternative molecule to the current antibiotics against resistant bacteria.

In the work of Buakaew et al., the effect of β-citronellol, identified in *Citrus hystrix* DC. Leaf, against *Candida albicans* was evaluated. The antifungal activity of β-Citronellol is widely recognized, but little is known about its mechanism of action. By a proteomic approach, Buakaew et al. identified three groups of *C. albicans* proteins regulated by β-citronellol. In particular, the expression of cell wall proteins, cellular stress response enzymes, and ATP synthesis-associated proteins were altered after β-citronellol treatments, thus inhibiting *C. albicans* growth and biofilm formation.

Identifying and characterizing new molecules of natural origin with promising antimicrobial properties represents a priority in response to the health emergency due to drug resistance.

The discovery of new antimicrobials, to be used alone or in synergy with conventional antibiotics, is of extreme importance for the treatment of pathologies caused by resistant microorganisms. As a result, being able to treat resistant bacteria-related infections, these natural antimicrobials could contribute to decreasing the risk of complications and death associated with such infections, besides drastically reducing the costs of treatments and health care.

For these reasons, we are confident that this article collection is a valid contribution to this Research Topic.

## Author contributions

RS, CG, and PF wrote the editorial draft. RS, CG, FD'A, and PF edited and finalized this manuscript. All authors contributed to the article and approved the submitted version.
